# Introduction of Telemedicine in a Prehospital Emergency Care Setting: A Pilot Study

**DOI:** 10.1155/2023/1171401

**Published:** 2023-03-23

**Authors:** Christophe Jobé, Pierre-Nicolas Carron, Pierre Métrailler, Jean-Marc Bellagamba, Alexandre Briguet, Line Zurcher, Fabrice Dami

**Affiliations:** ^1^Emergency Department, Lausanne University Hospital (CHUV), Lausanne, Switzerland; ^2^University of Lausanne, Lausanne, Switzerland; ^3^Helicopter Emergency Medical Services, Air Glacier, Sion, Switzerland; ^4^Emergency Medical Services, Dispatch Centre, State of Valais, Sion, Switzerland; ^5^Emergency Medical Services, Dispatch Centre, State of Vaud (Fondation Urgences-Santé), Lausanne, Switzerland

## Abstract

**Background:**

Advances in information and communication technology have led to telemedicine applications that could support paramedics in the prehospital field. In an effort to optimise the available resources like prehospital emergency physicians (PHP), the State Health Services of a Swiss state decided to launch a pilot study on the feasibility of using telemedicine in the prehospital emergency setting.

**Objective:**

The primary objective was to measure the number of missions completed without technical problems with remote PHP support through telemedicine (tele-PHP). The secondary objectives were to evaluate the safety of this protocol and to describe the actions and decisions that clinicians can make by using tele-PHP.

**Methods:**

This was a prospective observational pilot study on all missions involving the dispatch of ground PHP or tele-PHP. The severity score, dispatch criteria, actions, and decisions made by ground PHP and tele-PHP were collected.

**Results:**

PHP were dispatched simultaneously with an ambulance on 478 occasions, including 68 (14%) situations that started directly with tele-PHP. Among those situations, three had to be transformed into on-site PHP missions after the on-site evaluation by paramedics. Fifteen missions were cancelled by paramedics once they were on site, and six missions encountered a connection issue. Forty-four PHP missions that were dispatched simultaneously with paramedics were completed by tele-PHP only without any connection problems. Paramedics and PHP estimated that actions or decisions were provided by PHP in 66% of the on-site PHP missions and 34% of the tele-PHP missions.

**Conclusions:**

This is the first experience of tele-PHP regarding PHP dispatch in Switzerland. Despite the small number of missions carried out, tele-PHP could be used for well-selected situations to reduce the need for a PHP on site.

## 1. Introduction

Telemedicine has been known and used for many years. As defined by the World Health Organization, telemedicine is “the provision of healthcare services at distance with communication conducted between healthcare providers seeking clinical guidance and support from other healthcare providers (provider-to provider telemedicine); or conducted between remote healthcare users seeking health services and healthcare providers (client-to-provider telemedicine).” It has a very broad scope of application [1–3]. The specific issue studied in this work is the possible added value of bringing medical knowledge and decision-making capacity to paramedics on site, without having to dispatch prehospital emergency physicians (PHP). The current use of telemedicine includes the transmission of 12-lead electrocardiograms (ECG) to in-hospital physicians, an approach that has optimised the “door-to-balloon” time for ST elevation myocardial infarction (STEMI) [4]. Telemedicine has also provided rapid recognition and orientation capacities for patients presenting with suspicion of a stroke [5–8]. When trauma is added, these three topics account for 80% of the current literature. The impact of telemedicine on patients and healthcare providers in prehospital care is encouraging, but it is still understudied and in its nascent stage of development [9, 10].

In a trend to optimise the available resources such as PHP, the State Health Services of a Swiss state decided to launch a pilot study on the feasibility of using telemedicine in the prehospital emergency setting. While keeping the same dispatch criteria for PHP, they would only be sent on site (on-site PHP) in the most serious supposed conditions; otherwise, they would give advice at a distance (tele-PHP) to paramedics. The primary outcome of this study was to evaluate the feasibility of proposing remote PHP support as supportive care. The secondary outcomes were to evaluate the safety of the new dispatch protocol and to describe the actions and decisions provided by PHP and tele-PHP.

## 2. Method

### 2.1. Setting

The dispatch centre of the studied region, Central Valais, uses the Advanced Medical Priority Dispatch System (AMPDS™) version 13.3 to triage calls. Dispatchers are nurses, paramedics, or graduates in emergency dispatch. They manage more than 45,000 calls per year and dispatch about 18,000 ambulances. It is a three-tiered system: the dispatch centre, paramedics, and PHP dispatched by ground or helicopter. Historically, in this system, all delta and echo AMPDS codes have required dispatch of a PHP in support of the ambulance. Echo codes correspond mainly to cardiac arrest (09E01) or severe dyspnoea with risk of imminent death (11E01). Delta codes correspond to potential life-threatening emergencies with various initial symptoms ranging from dyspnoea (11D01) to polytrauma (17D05) and acute chest pain (19D04). For other codes, paramedics may ask for a PHP once on site if they deem it necessary. Following their first evaluation on site, paramedics may also cancel the PHP while they are on their way if they deem a PHP unnecessary.

In Switzerland, paramedics receive 3 years of specific education. They work under delegated protocols (intravenous and intraosseous lines; ECG; oxygen; resuscitation; and about 10 medications such as opiates, benzodiazepines, epinephrine, and naloxone). PHP are anaesthetists or emergency physicians.

The studied area is a sole semiurban PHP territory surrounded by high mountains and isolated small valleys. It is a very tourist-heavy area during the ski season, when the population grows from 100,000 to 200,000. This territory represents 25% of all missions of the state.

### 2.2. Study Design

This was a prospective observational pilot study. State Health Services first performed a retrospective analysis of all PHP missions (*n* = 5,044) incorporating the entire State of Valais territory from 2018 to 2019. PHP missions were rated according to the National Advisory Committee for Aeronautics (NACA) (Table 1). The NACA score is an eight-level scale to assess prehospital severity status; the score is defined by the most serious clinical state experienced at any given time during the mission. The NACA score is significantly correlated with survival [11]. Missions with a low severity code (≤3) are *a priori* considered as not needing a PHP on site and therefore potentially relevant for tele-PHP. Half of PHP missions had a NACA *score* ≤ 3 or were cancelled by paramedics after the first evaluation during the 2018-2019 observation period prior to the beginning of the study (*n* = 2,707, 54%); these results are comparable to studies conducted by neighbouring Swiss states [12, 13]. The AMPDS codes of each of these missions were analysed. After excluding all trauma codes often requiring a technical procedure by a PHP to obtain good analgesia (fracture reduction), all nontrauma delta codes with a NACA *score* ≤ 3 were considered suitable for tele-PHP in the dispatch system. All AMPDS codes selected to use tele-PHP are summarised in Table 2. Therefore, a potential reduction of 20%-30% of on-site PHP missions was estimated.

### 2.3. Study Population

All missions involving a PHP dispatch, according to the AMPDS criteria, from 15 July 2020 to 31 December 2021 were included. For safety reasons, the territory is reduced to a distance of 20 minutes by car around the ground physician base for the use of tele-PHP; so if the tele-PHP mission must be transformed into an on-site PHP mission, a PHP could reach the patient within a reasonable amount of time. If the estimated delay is over 20 minutes, tele-PHP is not proposed.

According to the new dispatch protocol, once a dispatch code proposes tele-PHP, the dispatcher calls the PHP. After a short explanation of the mission and discussion, the PHP considers the estimated risks and decides whether tele-PHP is appropriate or whether he/she favours going on site.

When a PHP is not primarily dispatched, once on site, the paramedics can ask for tele-PHP or on-site PHP if they deem it necessary. After the first evaluation, they can also cancel a PHP who has been primarily dispatched before their arrival on site.

Once the initial assessment is done, the paramedics start an audioconference with the tele-PHP. The tele-PHP turns on his/her tablet and can view the monitor remotely. Hence, the patient is managed by the paramedic and the PHP. This can be completed via telemedicine or the tele-PHP could decide to go on site. In addition to their standard monitor, all ambulances participating in the study were equipped with a Tempus ALS monitor by Philips™ (rented by the state), which can send data to the PHP via live streaming with the i2i ReachBak™ software. It allows the PHP to see “live” the scope with a three-lead ECG, oxygen blood saturation, blood pressure, and, when appropriate, a 12-lead ECG. As there is a lack of current legislation in the state on the use of video, the video function was not used. All PHP and paramedics received a half-day training session on how to use the monitor and the software.

During the study period, as a form of quality control, if a tele-PHP mission needed to be switched to an on-site PHP mission, the State Health Services evaluated whether the dispatch rule regarding the code needed to be changed.

### 2.4. Data Collection

The secure online software http://nowplay.service-now.com was used to collect the mission data, including the AMPDS dispatch code, the dispatch proposal from the system, the compliance of dispatchers and PHP with the AMPDS codes, and the NACA scores determined by the paramedics. At the end of the mission, paramedics and PHP completed a questionnaire on the actions and decisions made by the PHP either on site or remotely.

### 2.5. Statistics

Demographic and clinical characteristics are summarised using descriptive statistics, including frequencies for categorical variables. All data were entered into a Microsoft Excel spreadsheet.

### 2.6. Outcomes

The primary outcome was to describe the feasibility of proposing remote PHP support as supportive care instead of an on-site PHP dispatched at the same time as paramedics. Feasibility was measured by the number of missions completed without technical problems. The secondary outcomes were to evaluate the safety of the dispatch protocol, defined as the absence of missions with a high NACA severity score (>3) that did not benefit from an on-site PHP and the absence of the need to switch from tele-PHP to an on-site PHP mission after the first evaluation to evaluate the compliance of the dispatchers and PHP with the AMPDS dispatch proposal (the number of overruled missions), and finally to describe the actions and decisions made by tele-PHP as well as on-site PHP.

### 2.7. Ethical Aspects

The local ethical committee exempted this work from the need to submit a formal request, as it was considered to not be under the scope of Swiss human research legislation (req. 2022-00111).

## 3. Results

**Figure 1:**
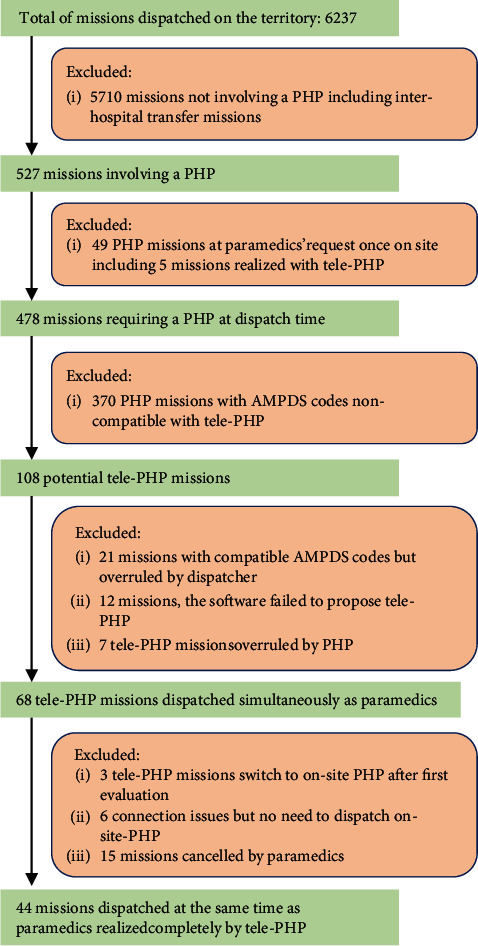
Flow chart.

During the study period, the dispatch centre handled 6,237 missions (including interhospital transfer missions) in the studied PHP territory. A total of 527 (8%) missions involved a PHP, 478 according to the dispatch code and 49 at the paramedics' request once on site (Figure 1).

Among the 478 PHP dispatched simultaneously, 108 (22%) had an AMPDS code compatible with tele-PHP. Of those 108 potential tele-PHP missions, dispatchers overruled and transformed tele-PHP into on-site PHP on 21 occasions (the symptoms were chest pain, unconsciousness, overdose, specific illness, and respiratory issues), and PHP overruled 7 of 75 tele-PHP propositions and went on site. The codes of overruled missions are presented in Table 3. On those seven missions, three were cancelled by paramedics before the arrival of a PHP, two had a NACA score of 2, one had a NACA score of 4, and one had a NACA score of 5. Among those 108 potential tele-PHP missions, the software failed to propose tele-PHP on 12 occasions for unknown reasons (Figure 1).

Sixty-eight tele-PHP missions (14% of the total PHP missions) were dispatched simultaneously with paramedics. Among those tele-PHP, three required on-site PHP after the paramedics' first evaluation on site. The main symptoms were respiratory issues (NACA *score* = 6) and two cases of chest pain (NACA *score* = 4 and 6) (Table 3). Six missions encountered technical problems: either the Tempus ALS monitor by Philips™ was unable to transmit data via the mobile network or the PHP was unable to connect to the online software with his/her tablet. Paramedics used a standard phone to complete the missions. Fifteen PHP were cancelled once paramedics were on site.

Finally, 44 missions were completed with tele-PHP following the dispatch decision, and five more were completed with tele-PHP at the paramedics' request once on site. Hence, there were 49 tele-PHP missions performed (11% of the total 458 PHP missions performed, excluding cancelled missions).

Among the 68 tele-PHP missions dispatched at the same time as paramedics, 11 had a NACA *score* > 3 (16%). No patient died during a tele-PHP mission.

Of the 370 on-site PHP missions with AMPDS codes not compatible with tele-PHP, 179 (48%) had a NACA *score* > 3. The details of the results for all missions involving a PHP according to the NACA score are available in Table 4.

Regarding the 49 missions performed with tele-PHP, according to the survey, paramedics and PHP estimated that actions or decisions were made by PHP on 17 missions (34%), mainly to provide help with the diagnosis and patient orientation. The details of actions and decisions made by PHP are available in Table 5. Regarding the 403 on-site PHP missions performed, paramedics and PHP estimated that actions or decisions were made by the PHP on 267 occasions (66%), mainly regarding therapy, providing help with the diagnosis, and completing a death certificate (Table 5).

## 4. Discussion

During the study period, 68 tele-PHP were simultaneously dispatched with paramedics. Fifteen missions were cancelled by paramedics once on site, three missions required a physician on-site at the paramedics' request, and six other missions encountered a technical issue. This last point demonstrates that the feasibility of tele-PHP is not yet satisfactory. Although the severity of this case mix was low (11 of the 68 missions (16%) had a NACA *score* > 3), three missions required a switch to an on-site PHP. Thus, the objective of no conversion from tele-PHP to on-site PHP was not reached. This outcome may require a revision of the AMPDS codes that allow tele-PHP.

There were 28 missions overruled by the PHP or dispatcher, which represent 26% of the AMPDS codes compatible with tele-PHP. The right of the PHP to modify the assignment of missions is present in the protocol, which allows for flexibility, but it is subjective and dependent on the PHP. However, dispatchers overruled the protocol by modifying the mission assignments, an outcome that was not expected. The large number of these events (21 missions or nearly 20% of the compatible AMPDS codes) represents a major limitation of this study. Considering missions with AMPDS codes suitable for tele-PHP, there was a higher percentage of missions with a NACA *score* > 3 overruled by the dispatcher (46%) than missions with a NACA *score* > 3 completed with tele-PHP (16%). If all those potential tele-PHP missions had been carried out with tele-PHP, then the estimate of performing 20%-30% of all PHP missions with tele-PHP would have been achieved. This finding is similar to the retrospective analysis conducted by the Valais State Health Services (2018-2019) for AMPDS code assignment, as well as two studies in neighbouring states, namely, Vaud and Geneva, owing to the use of PHP with good sensitivity to life-threatening situations but with poor specificity [12, 13]. This would allow for the optimisation of available resources, especially PHP, who are costly to educate and sometimes difficult to recruit, but who are also needed within emergency rooms, where paramedics and telemedicine cannot replace them.

The State Health Department performed a continuous quality and safety evaluation of the protocol. They looked especially at tele-PHP missions that were switched to an on-site PHP mission and evaluated the reasons. During the first 6 months of the study, six AMPDS codes were changed from tele-PHP to on-site PHP: difficulties breathing at rest (06D02), convulsions (12D01 and 12D02), diabetic issues (13D01), and unconscious without specification or with efficient breathing (31D02 and 31D03). Conversely, one code—chest pain (10D02)—was changed from on-site PHP to tele-PHP. These status changes were evaluated once a month during the follow-up study. Because safety was a priority, it was easy to change codes between on-site and tele-PHP; therefore, some AMPDS codes were found with both assignments, which may bias some results. These were not excluded from the study as it remains an observational work.

A difficulty encountered during this project was the number personnel involved: 33 paramedics and 41 PHP, with a maximum of nine tele-PHP missions per PHP. Therefore, they did not get the opportunity to become accustomed to this new practice even after 18 months. Their initial acceptance of the project was low, and their number also made it difficult to collect data, requiring a considerable number of reminders to complete the questionnaire. The reluctance of PHP with respect to the project is due to the change in work habits it implies. PHP are not used nor trained to make medical decisions when they are not on site. Moreover, the project required data collection on a specific tool, which implied that they would have to do more work. The fear of endangering patients and the decrease of on-site missions were also disadvantages for them. On the other hand, the study took place after the beginning of the COVID-19 pandemic, which improved acceptance of the use of telemedicine. Indeed, telemedicine became widely accepted during the first containment [2, 3, 14]. Reducing the exposure of PHP to COVID-19 for unnecessary situations was an additional reason for the State of Valais to start this study.

The previously described added value of an on-site PHP mainly concerns cardiac arrest and major trauma [15, 16]. PHP may also treat patients more easily and leave them on site when possible. This potentiality would bring some relief to the emergency department [17] or provide emergency palliative care and therefore relief to patients, avoiding unnecessary transport to the hospital [18]. Regarding actions performed and decisions made by PHP during a mission, according to paramedics and PHP, whether remote or on site, the results show that the higher the severity of the mission (NACA score), the greater their contribution, which is not a surprise. Of note, when a PHP was on site in this case mix, their added value was mainly to help with the diagnosis and therapeutic decisions, rather than to help with technical procedures or legal aspects, as was hypothesised. Based on this outcome, we believe that it would be possible to widen the indications for tele-PHP.

A research team in Aachen (Germany) is currently one of the most advanced regarding telemedicine. However, their model is quite different, with a PHP dedicated to tele-PHP and located within the dispatch centre. Their first encouraging studies were based on analgesic care, STEMI, and hypertensive crisis [19–23]. After 3 years of utilisation, they demonstrated the safety and technical performance of telemedicine [24]. The main difference with this study is that the first use of tele-PHP in Aachen was for missions that, in our system, would not require an on-site PHP to be dispatched because paramedics would have sufficient autonomy in these situations. Our study included AMPDS codes for tele-PHP corresponding to disorders such as altered consciousness, chest pain, and shortness of breath, which was not the case in the Aachen studies.

Finally, the use of video (for dispatchers or PHP) was not part of the study design and was therefore not available. Subjectively, PHP did not feel that their attitudes would have been different if they had had access to video. In their pilot study in South Carolina (USA), Al Kasab et al. [25] suggested that in cases of suspected stroke, video could add value over audio alone. In contrast, the use of video in a retrospective study in Aachen (Germany) and in a systematic review did not significantly improve diagnostic support [26, 27].

## 5. Limitations

There are three major limitations to this study. First, to analyse the actions or decisions of the PHP, the questionnaires given to the paramedics and PHP had to be merged. Second, for safety reasons, State Health Services modified the dispatch protocols (tele-PHP vs. on-site PHP) during the observation, which affected the results. Finally, several technical issues were encountered by paramedics while using the Tempus ALS monitor.

## 6. Conclusion

This was the first experience of tele-PHP in Switzerland. Despite the small number of missions carried out, its use for well-selected situations is possible. It allows for a reduction in the need for an on-site PHP and offers the potential for even more on-site PHP missions to be reduced. This approach should now be tested in other settings.

## Figures and Tables

**Table 1:** Modified National Advisory Committee for Aeronautics (NACA) score used in the State of Valais.

NACA 0	No injury or disease
NACA 1	Injuries/diseases without any need for acute physician care
NACA 2	Injuries/diseases requiring examination and therapy by a physician, but hospital admission is not indicated. Including: large contusions, finger and toe fracture, second-degree burn (10%-20% of body surface), exhaustion without hypothermia
NACA 3	Injuries/diseases without acute threat to life but requiring hospital admission. Including: maxillofacial trauma, wound with vascular/neurological impact, third-degree burn (10%-20%), hypoglycaemia without coma, transient ischaemic attack, supraventricular arrhythmia with conserved haemodynamics, right iliac fossa pain syndrome, hypothermia stage I, second-degree burn (20%-30%), isolated limb fracture (femur excluded)
NACA 4	Injuries/diseases which can possibly lead to deterioration of vital signs. Including: open skull fracture, hypothermia stage II, suspicion of acute coronary syndrome, suspicion of ectopic pregnancy/placenta praevia
NACA 5	Injuries/diseases with acute threat to life. Including: head trauma *GCS* < 8, heart infarct, bradycardia (<30/min), tachycardia (>180/min), complete heart block, eclampsia, hypothermia stage III, haemodynamic shock, multiple rib fractures, acute dyspnoea, pulmonary oedema
NACA 6	Injuries/diseases transported after successful resuscitation. Including: chest trauma with severe dyspnoea, aortic rupture, total airway obstruction, central apnoea, emergency external pacing, cardiac arrest (ventricular fibrillation or asystole from any cause)
NACA 7	Lethal injuries or diseases (with or without resuscitation attempts)
NACA 9	Cancelled mission

GCS: Glasgow Coma Scale.

**Table 2:** Advanced Medical Priority Dispatch System (AMPDS) codes selected to use remote prehospital emergency physician support through telemedicine (tele-PHP) during this study.

Abdominal pain	01D00	01D01	01D02			
Allergic reaction/poisoning	02D00	02D01	02D04			
Back pain (atraumatic or nonrecent trauma)	05D00	05D01	05D02			
Respiratory issues	06D01	06D03	06D04	06D05		
Carbon monoxide/inhalation/toxic products	08D00	08D03	08D05	08D06		
Chest pain	10D00	10D01	10D02	10D03	10D04	10D05
Suffocation	11D00					
Convulsions	12D00	12D04				
Diabetic issues	13D00					
Eye issues	16D00	16D01				
Cardiac issues	19D00	19D01	19D03	19D04		
Cold or heat exposure	20D00	20D01	20D02			
Overdose/poisoning (ingestion)	23D00	23D01	23D02			
Psychiatric/behaviour issues/suicide attempt	25D00	25D01	25D02			
Specific illness (diagnosis known)	26D00	26D01				
Unconscious	31D04					

**Table 3:** Advanced Medical Priority Dispatch System (AMPDS) codes of remote prehospital emergency physician support through telemedicine (tele-PHP) missions overruled by dispatcher and PHP and codes of tele-PHP missions required PHP once paramedics were on site.

Missions overruled by dispatchers	Respiratory issues	6D01	6D04
	Chest pain	10D04
	Unconscious	31D04
	Overdoses	23D01
	Specific illness	26D01

Missions overruled by PHP	Respiratory issues	6D01	6D04
	Chest pain	10C00	10C03	10D02
	Psychiatric/behaviour issues/suicide attempt	25D00
	Unconscious	31D04

On-site PHP dispatch after starting tele-PHP	Respiratory issues	6D02
	Chest pain	10D04

**Table 4:** National Advisory Committee for Aeronautics (NACA) score of all missions involving a prehospital emergency physician (PHP) or a telemedicine with a prehospital emergency physician (tele-PHP).

Results	NACA score			Total
	≤3	>3	=9^∗^	
Missions dispatched				6237
Missions involving a PHP (tele or on site) including at the paramedics' request	228: 43%	230: 43%	69: 14%	527
(i) On-site PHP dispatched after first evaluation at the paramedics' request	18	21	5	44
(ii) Tele-PHP dispatched after first evaluation at the paramedics' request	2	3	0	5
Missions requiring a PHP at dispatch	208: 43%	206: 43%	64: 14%	478
(i) PHP missions with AMPDS codes non-compatible with tele-PHP	146	179	45	370
Potential tele-PHP missions	62: 57%	27: 25%	19: 18%	108
(i) Tele-PHP compatible AMPDS codes but overruled by the dispatcher	9	11	1	21
(ii) The software failed to propose tele-PHP	9	3	0	12
(iii) Tele-PHP missions overruled by PHP	2	2	3	7
Tele-PHP missions dispatched at the same time as paramedics	42: 62%	11: 16%	15: 22%	68
(i) On-site PHP dispatch after starting tele-PHP	0	3	0	3
(ii) Connection issues but no need to dispatch on-site PHP	6	0	0	6
(iii) Mission cancelled by paramedics	0	0	15	15
Tele-PHP missions dispatched at the same time as paramedics and completely performed by tele-PHP	36: 82%	8: 18%	0: 0%	44

^∗^NACA 9: PHP mission cancelled; AMPDS: Advanced Medical Priority Dispatch System.

**Table 5:** Details of prehospital emergency physician (PHP) actions/decisions.

PHP missions	Tele-PHP (49^∗^)	On-site-PHP (403^∗∗^)	Total (452)
PHP actions/decisions°	17 (34%)	267 (66%)	284 (63%)
Therapy (*n*; percentage)°	6 (12%)	122 (30%)	128 (28%)
(i) IV hydration	0	7	7
(ii) Toxic antagonist	0	1	1
(iii) Painkillers	0	14	14
(iv) Antiarrhythmic	0	4	4
(v) Antihypertensive	0	4	4
(vi) Antiplatelet anticoagulation	1	11	12
(vii) Sedation	0	20	20
(viii) Bronchodilator	1	8	9
(ix) Vasopressor	0	19	19
(x) Glucose	0	4	4
(xi) Other treatment	4	17	21
Medical decision°	15 (30%)	122 (30%)	137 (30%)
(i) ECG analysis	2	22	24
(ii) Diagnosis	13	90	103
(iii) Patient orientation	0	10	10
Technical gesture°	1 (2%)	66 (16%)	67 (15%)
(i) Ventilation	0	10	10
(ii) Intubation	0	21	21
(iii) Defibrillation	0	2	2
(iv) Pneumothorax exsufflation	0	1	1
(v) External pacing	0	2	2
(vi) Cardioversion	0	2	2
(vii) Thoracotomy	0	0	0
(viii) Leadership	1	29	30
Legal aspect°	0 (0%)	72 (18%)	72 (16%)
(i) Death certificate	0	46	46
(ii) Leave the patient on site	0	11	11
(iii) Privation of liberty	0	15	15
(iv) Decision not to reanimate a patient	0	23	23

^∗^49 tele-PHP missions performed including five tele-PHP missions dispatched after first evaluation at the paramedics' request. Cancelled missions and missions with connection issues (6) are excluded. ^∗∗^403 on-site PHP mission performed including 44 on-site PHP missions dispatched after first evaluation at the paramedics' request and the three tele-PHP switched to on-site PHP mission. Cancelled missions are excluded. °Missions can have more than one added value such as *do not attempt to resuscitate* or *termination of resuscitation* and establishing a death certificate. ECG: electrocardiogram; IV: intravenous.

## Data Availability

Raw data were generated at OCVS. Derived data supporting the findings of this study are available from the corresponding author (CJ) on request.

## Abbreviations

AMPDS: Advanced Medical Priority Dispatch System
NACA: National Advisory Committee of Aeronautics
PHP: Prehospital emergency physician
Tele-PHP: Prehospital emergency physician working with telemedicine.
